# Green pepper fruits counting based on improved DeepSort and optimized Yolov5s

**DOI:** 10.3389/fpls.2024.1417682

**Published:** 2024-07-16

**Authors:** Pengcheng Du, Shang Chen, Xu Li, Wenwu Hu, Nan Lan, Xiangming Lei, Yang Xiang

**Affiliations:** College of Mechanical and Electrical Engineering, Hunan Agricultural University, Changsha, China

**Keywords:** DeepSORT, deep learning, green pepper, fruit counting, track tracking

## Abstract

**Introduction:**

Green pepper yield estimation is crucial for establishing harvest and storage strategies.

**Method:**

This paper proposes an automatic counting method for green pepper fruits based on object detection and multi-object tracking algorithm. Green pepper fruits have colors similar to leaves and are often occluded by each other, posing challenges for detection. Based on the YOLOv5s, the CS_YOLOv5s model is specifically designed for green pepper fruit detection. In the CS_YOLOv5s model, a Slim-Nick combined with GSConv structure is utilized in the Neck to reduce model parameters while enhancing detection speed. Additionally, the CBAM attention mechanism is integrated into the Neck to enhance the feature perception of green peppers at various locations and enhance the feature extraction capabilities of the model.

**Result:**

According to the test results, the CS_YOLOv5s model of mAP, Precision and Recall, and Detection time of a single image are 98.96%, 95%, 97.3%, and 6.3 ms respectively. Compared to the YOLOv5s model, the Detection time of a single image is reduced by 34.4%, while Recall and mAP values are improved. Additionally, for green pepper fruit tracking, this paper combines appearance matching algorithms and track optimization algorithms from SportsTrack to optimize the DeepSort algorithm. Considering three different scenarios of tracking, the MOTA and MOTP are stable, but the ID switch is reduced by 29.41%. Based on the CS_YOLOv5s model, the counting performance before and after DeepSort optimization is compared. For green pepper counting in videos, the optimized DeepSort algorithm achieves ACP (Average Counting Precision), MAE (Mean Absolute Error), and RMSE (Root Mean Squared Error) values of 95.33%, 3.33, and 3.74, respectively. Compared to the original algorithm, ACP increases by 7.2%, while MAE and RMSE decrease by 6.67 and 6.94, respectively. Additionally, Based on the optimized DeepSort, the fruit counting results using YOLOv5s model and CS_YOLOv5s model were compared, and the results show that using the better object detector CS_YOLOv5s has better counting accuracy and robustness.

## 1 Introduction

Green pepper, a significant commercial crop, can undergo multiple harvests based on fruit maturity during the growing season from June to November (Stein et al., 2016), and pepper yields exhibit considerable variation across different harvesting periods. Consequently, pre-harvest estimation of green pepper yield can significantly aid in optimizing harvest processes, labor management, transportation, and storage conditions (He et al., 2022). Currently, green pepper yield estimation predominantly depends on manual sampling, a time-consuming and labor-intensive method (Aggelopoulou et al., 2010; Wulfsohn et al., 2012). Recent advancements in machine vision and deep learning have demonstrated substantial potential in enhancing fruit yield estimation accuracy (Ren and Yang, 2016). This integration offers an effective solution for the yield estimation of green peppers.

Fruit yield can be reflected laterally by the quantity (Teixidó et al., 2012; Zhang Y. et al., 2022; Payne et al., 2013). Dorj et al. (2017) and Malik et al. (2016) implemented distinct image thresholding techniques to segregate citrus fruits from the background. This approach was used for detecting and counting citrus fruits in single images. Tu et al. (2020) and Behera et al. (2021) utilized the Faster-RCNN convolutional neural network for fruit detection and counting in single images.

The correlation between the number of fruits in a single image and the count estimated by the algorithm is approximately 95%. However, counting individual scattered fruits in single images may not accurately reflect the total count of fruits in the entire orchard. Therefore, when counting fruits in an orchard, it is common to use interval image sampling to reflect the overall situation of the entire orchard. For example, Song et al. (2014) counted peppers on densely planted trees by capturing images at 5cm intervals, thereby obtaining multi-view representations of the same fruit. They employed the ‘bag of words’ model for pepper detection in individual images and identified the same peppers across multiple images by analyzing coordinate shifts, thus minimizing repeat counts. The experimental data revealed that the correlation coefficient between manual and automated pepper counting methodologies stood at 74%. It can be seen that although Y. Song et al. tried to reduce the repeat count by analyzing the peppers in adjacent images, the interval image sampling still caused the problem of the repeat count (Liu et al., 2019). To reduce such problems, when using interval sampling to count apples and oranges, Xia et al. (2022) deployed CenterNet for the detection of fruits and developed a patch-matching model to match the same fruit in adjacent images. The experimental results indicated a correlation between manual and algorithm counting of apples and oranges at 97.37% and 95.62% respectively. However, the patch-matching model necessitated a brute-force comparison of all patches, resulting in an average processing time of 5.33 minutes per image sequence.

To achieve rapid and accurate automatic fruit counting while reducing duplicate counts, many researchers employ multi-object tracking algorithms on sequences of fruit videos. These algorithms detect fruits in video sequences and assign a unique ID to each fruit, enabling the counting of fruits based on the number of IDs. Gao et al. (2022) utilized the YOLOv4-tiny algorithm in conjunction with the CSR-DCF algorithm for the detection and tracking of apples. This approach achieved a counting accuracy of 91.49% and enabled fruit tracking at speeds of 2–5 fps on a CPU. Similarly, Vasconez et al. (2020) compared the influence of multiple target detection algorithms on tracking and counting based on the Bayesian multi-target tracking algorithm. They found that, under the same tracking algorithm, object detection algorithms with higher detection performance often resulted in better counting accuracy. Additionally, they discovered that when processing fruit video sequences, the multi-object tracking required only 10 milliseconds per frame, and the average counting accuracy for different types of fruits reached 90%. While tracking fruits for counting can yield good results, the dense growth of fruit trees often leads to fruits being obscured by each other or by foliage. Fruits may disappear and reappear in video sequences at different time intervals (Li X. et al., 2022; Wu et al., 2023), significantly increasing the difficulty of fruit tracking. Therefore, to improve the accuracy of fruit counting, Gao F. et al. (2021) optimized the Hungarian matching algorithm based on the YOLOv4-tiny algorithm. This enhancement strengthened the association of the same apples across different time intervals in video sequences. Ultimately, they achieved an average counting accuracy of 81.94% for apples in the orchard. Similarly, Zhang W. et al. (2022) improved the YOLOv3 algorithm and SORT (Simple Online and Realtime Tracking) algorithm simultaneously to count citrus fruits in the field. By combining an efficient object detection algorithm with an accurate object tracking algorithm, they aimed to prevent duplicate counting caused by complex fruit occlusion. The method demonstrated a Mean Absolute Error of 8.1% and a Standard Deviation of 8% in orange counting.

Accurate fruit detection is imperative for accurate tracking (Mccool et al., 2016). The integration of precise object detection and efficient multi-object tracking algorithms makes automated fruit counting through video sequences feasible (Zhang W. et al., 2022). The detection of green peppers presents significant challenges due to their dense distribution on plants, severe occlusion by branches and leaves, and color similarity to the background. Traditional visual detection algorithms struggle to achieve both high precision and recall rates in green pepper detection (Stein et al., 2016). Convolutional Neural Networks (CNNs) have become the mainstream approach for feature extraction in green pepper detection. Li X. et al. (2021) proposed a detection model targeting the scale variation problem of green peppers based on YOLOv4-tiny. This model efficiently detects small green peppers, as well as highly overlapping and occluded ones, achieving a detection accuracy of 95.11%. The following year, Wang et al. (2022) balanced the parameter count and detection accuracy of the YOLOv5s model to detect small chili peppers. They achieved an mAP (mean Average Precision) value of 95.46% for this task. Cong et al. (2023) introduced a Swin Transformers attention mechanism into the Mask R-CNN model for effective segmentation of green peppers under strong lighting, overlapping instances, and heavy foliage occlusion, achieving an average detection accuracy of 98.1%. In addition, Wei et al. (2023) studied the influence of different light intensity and light Angle on the recognition and positioning of green pepper based on the YOLOv5s model. The study showed that the detection effect of green pepper was the best under the illumination Angle of 90°, and the mAP value was 97.3%.

Based on the research mentioned above, this paper proposes a novel method for automatic counting of green pepper fruits based on object tracking, aimed at facilitating rapid and accurate estimation of green pepper yields. Effective object detection and precise object tracking are crucial for achieving high counting accuracy. Therefore, this paper implements the following tasks:

### 1.1 Improvement of YOLOv5s model with lightweight design and attention mechanism

Enhance the YOLOv5s model by incorporating the concepts of lightweight model design and attention mechanisms. Adopt a Slim-Neck structure to maintain model accuracy while reducing computational complexity and enhancing detection speed. Integrate channel attention modules and spatial attention modules to improve feature perception and extraction of green peppers at different locations within images.

### 1.2 Optimization of DeepSort algorithm for green pepper tracking

Address the issue of double counting caused by ID switches during green pepper tracking by optimizing the matching mechanism within the DeepSort algorithm. These adjustments aim to reduce ID switching events, particularly those triggered by significant changes in green pepper motion characteristics.

### 1.3 Implementation of track post-processing method

Use a track post-processing method to optimize green pepper tracks, thereby minimizing double counting to the maximum extent possible.

## 2 Methods

**Figure 1:**
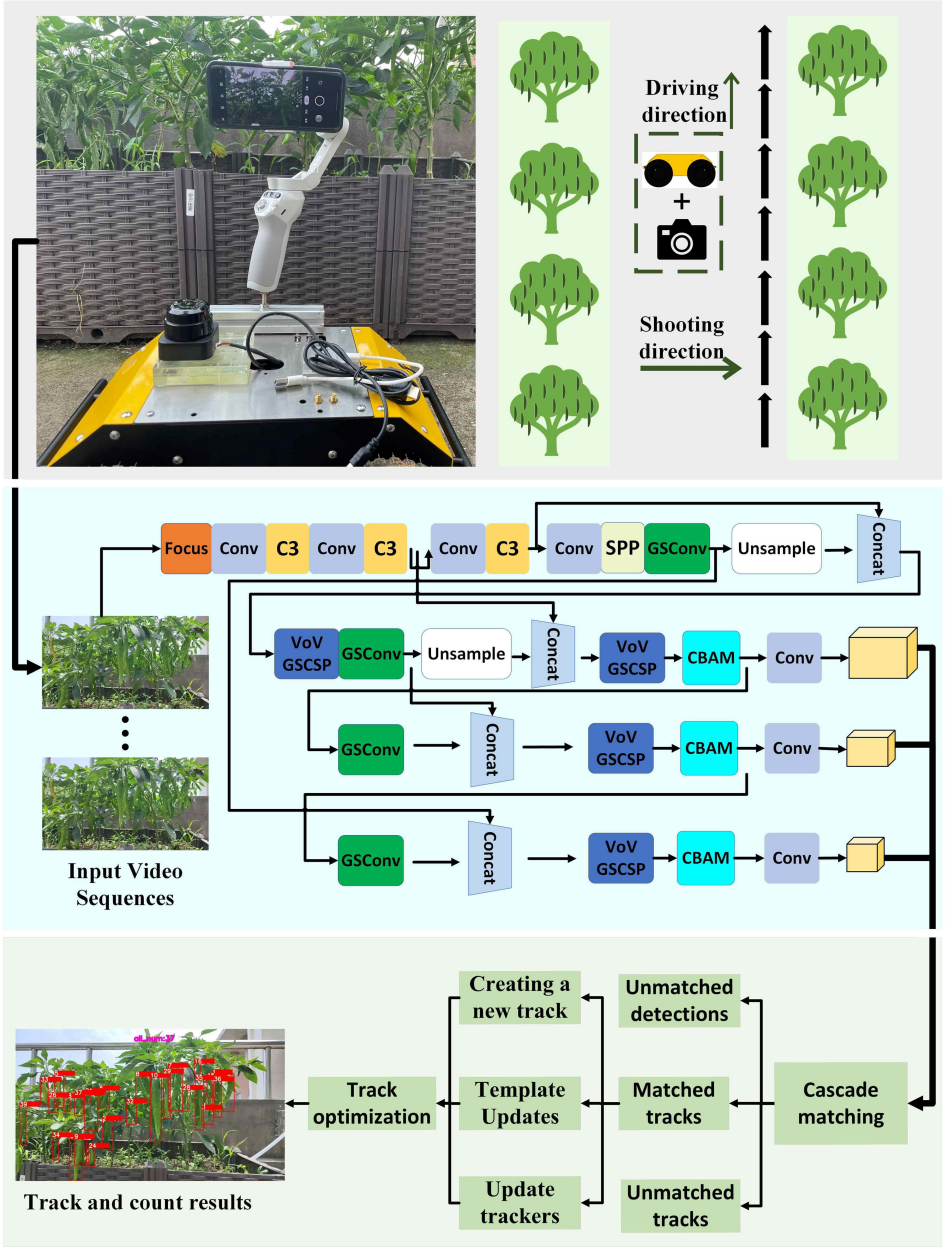
Roadmap of counting method.

The enhanced DeepSort multi-target tracking algorithm is integrated with the optimized YOLOv5s target detection algorithm for the counting of green peppers. The optimized YOLOv5s algorithm rapidly identifies green pepper fruits within the video sequence. Subsequently, the data about detected fruits are inputted into the enhanced DeepSort algorithm. This algorithm associates identical fruits and allocates a distinct ID to each one throughout the video sequence. Ultimately, the counting of green peppers is achieved by tallying the unique IDs assigned to each fruit. The technological roadmap for this counting method is depicted in **Figure 1**.

### 2.1 Optimized YOLOv5s object detection algorithm

The YOLOv5s algorithm, a one-stage detection framework, adeptly balances detection speed with accuracy. Its architecture comprises four primary components: the Input Layer, Backbone, Neck, and Prediction Head, as shown in **Supplementary Figure 1**. Within the Input Layer, green pepper images are resized to 640×640 pixels using adaptive picture scaling, ensuring training speed while maintaining model accuracy. Additionally, the Mosaic data augmentation method (Bochkovskiy et al., 2020) is employed, where four images are randomly scaled, cropped, and arranged together to enrich the dataset and enhance the model’s generalization ability. The Backbone network consists of structures such as Focus, BottleneckCSP (Redmon and Farhadi, 2018), and SPP (Spatial Pyramid Pooling) (He et al., 2014), which are used to extract target features from the input image. The Neck utilizes a network structure that integrates FPN (Feature Pyramid Network) (Lin et al., 2017) and PAN (Path Aggregation Network) (Liu et al., 2018) to aggregate high-level and low-level features, reducing the loss of target features due to spatial compression and channel expansion. This integration helps preserve important features across different scales and resolutions, improving the overall effectiveness of the feature extraction process. The Prediction Head utilizes operations such as NMS (Non-Maximum Suppression) (Bodla et al., 2017) to determine the best bounding boxes and applies other techniques for refining predictions. Additionally, it outputs three different-sized feature maps to predict targets of various sizes. This approach allows for comprehensive object detection across different scales within the input image.

**Figure 2:**
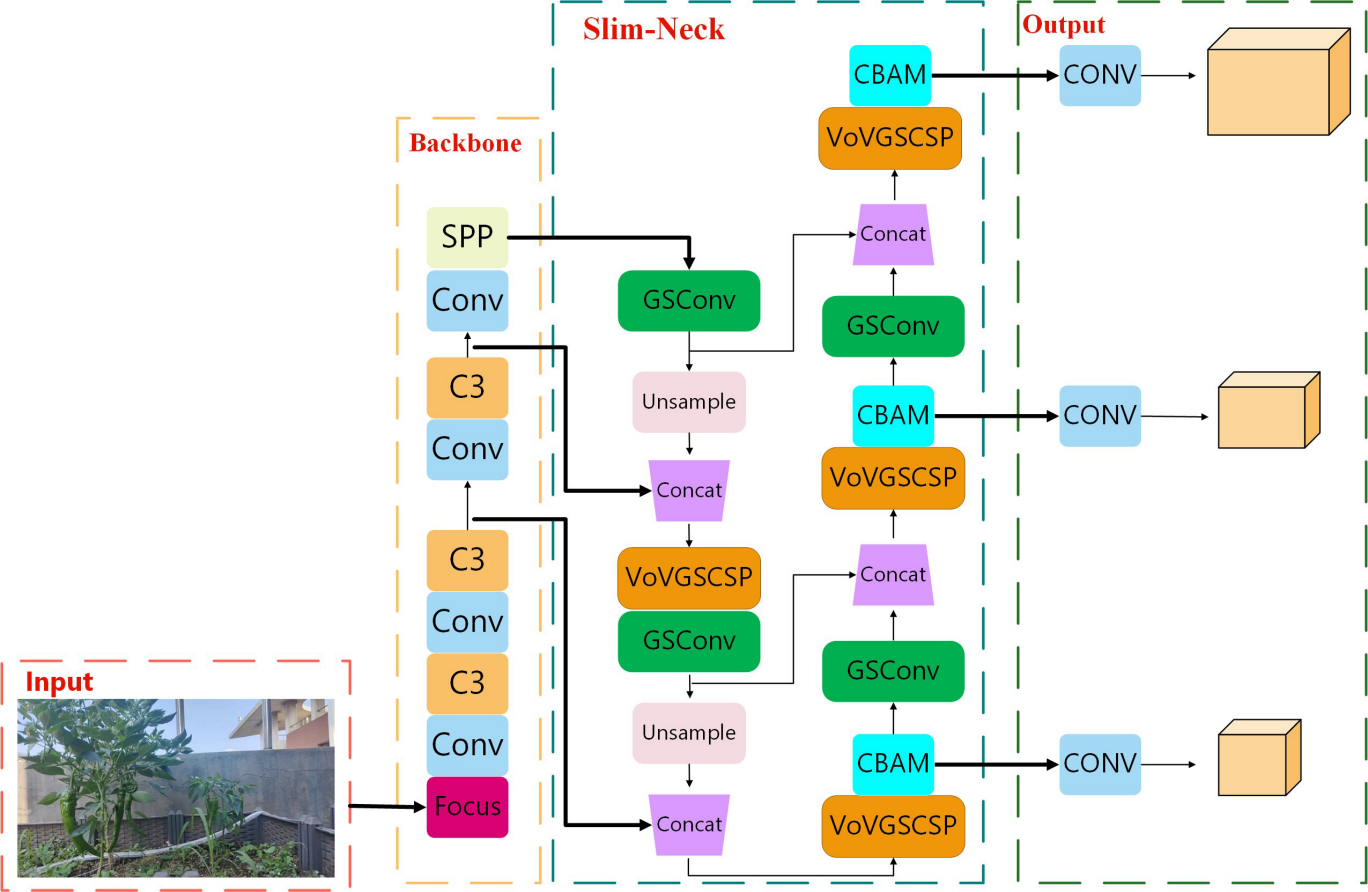
Network structure of the CS_YOLOv5s algorithm.

In the process of identifying green peppers, the similarity in color between fruits and leaves, as well as their overlapping and occlusion by branches and leaves, all affect green pepper fruit recognition. The accuracy and speed of green pepper fruit recognition also influence the subsequent tracking of it. Therefore, this paper proposes the CS_YOLOv5s model based on the YOLOv5s model for green pepper detection. Firstly, the Slim-Neck combined with GSConv (Li H et al., 2024) is used to optimize the Neck part of the YOLOv5 model, achieving a better balance between detection accuracy and speed. GSConv integrates DWconv and Conv modules to accelerate prediction calculations while minimizing the loss of semantic information during spatial compression and channel expansion of feature maps. Simultaneously, a cross-stage partial network module VoV-GSCSP, designed using a one-shot aggregation method, is employed for effective information fusion between feature maps at different stages. This reduces the complexity of computations and network structures while maintaining sufficient accuracy. To mitigate the interference of green pepper branches and leaves on fruit recognition, the CBAM attention mechanism is introduced at the connection between the Neck part and the Prediction Head of the YOLOv5s model. This enhances the perception of green pepper fruit features at different positions, improves the model’s feature extraction capability, and increases the attention to green pepper fruit features. **Figure 2** illustrates the network structure of the optimized YOLOv5s algorithm.

#### 2.1.1 CBAM attention mechanism

**Figure 3:**
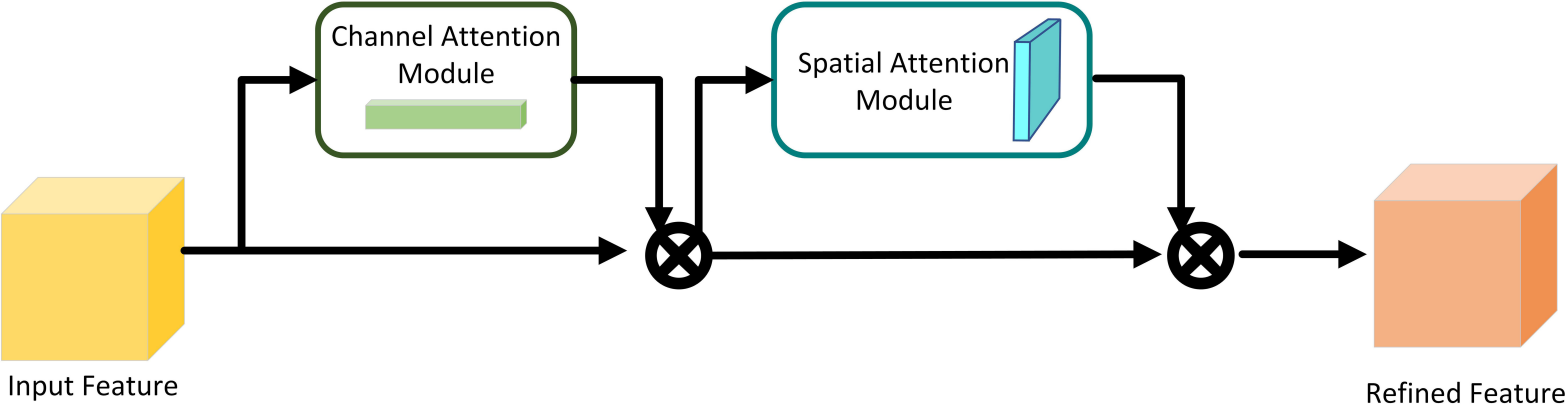
CBAM structure diagram.

CBAM (Woo et al., 2018) (Convolutional Block Attention Module) represents an attention mechanism tailored for image processing, encompassing two sub-modules: Channel Attention (Lu and Hu, 2022) and Spatial Attention (Gao C. et al., 2021). The structural layout of CBAM is depicted in **Figure 3**. This module enhances green pepper recognition by further extracting features from channels and spatial locations, effectively suppressing interference information and accentuating significant locations.

#### 2.1.2 Slim-neck combined with GSConv

**Figure 4:**
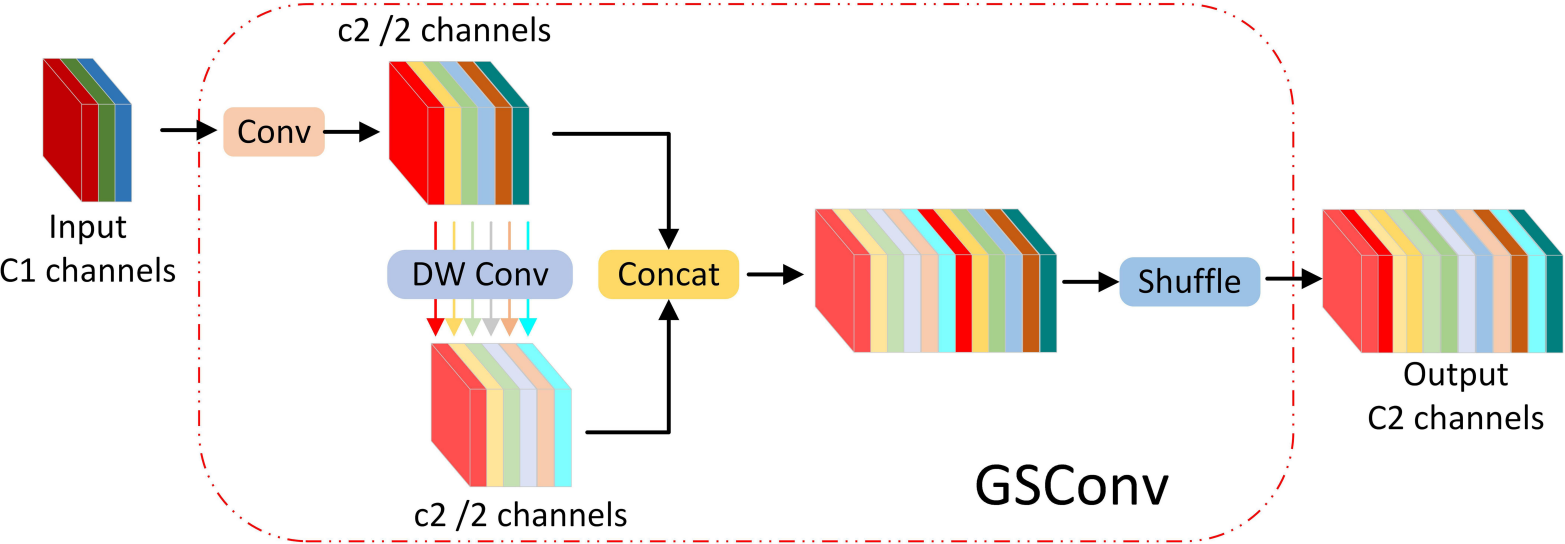
GSConv structure diagram.

**Figure 5:**
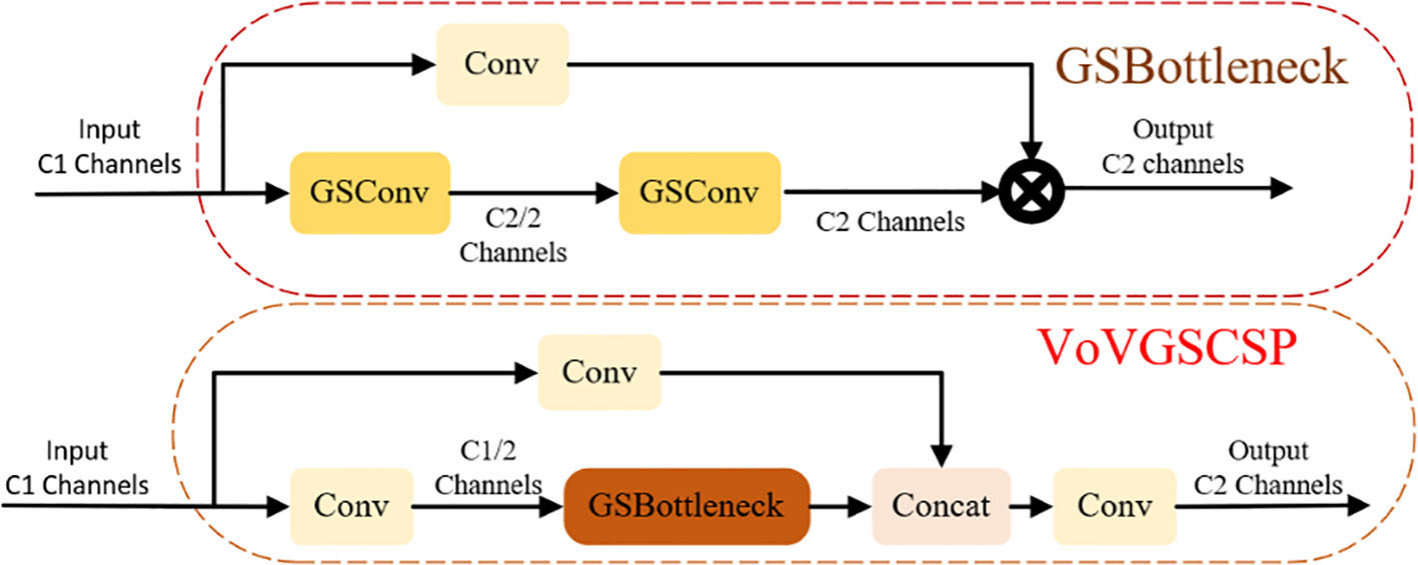
Slim-Neck structure diagram.

Within the Neck layer of YOLOv5s, the integration of Slim-Neck with GSConv has yielded notable results in the field of automotive autonomous driving. To form the Slim-Neck network structure, the C3 module in the Neck layer is substituted with the VoVGSCSP module. This alteration enhances the model’s generalization capabilities and concurrently reduces the number of channels and parameters. Within the Slim-Neck structure, the GSConv module replaces the standard Convolution (Conv) module. GSConv fuses Standard Convolution (Zhou et al., 2022) (SC) with Depth-wise Separable Convolution (Hossain et al., 2021) (DSC). Through a Shuffle structure, information from SC is intermingled with that from DSC, ensuring both the stability of the model’s performance and a reduction in parameters. The combined Slim-Neck and GSConv network structure effectively minimizes the number of parameters while amplifying the model’s feature extraction capabilities. **Figures 4** and **5** illustrate the structures of GSConv and Slim-Neck, respectively.

### 2.2 DeepSort multiple target tracking algorithm

The DeepSort multi-target tracking algorithm comprises four key components: the Kalman filter algorithm (Li et al., 2015), the Hungarian algorithm, the Reid model, and Cascade matching. In the following frame, the Kalman filter algorithm estimates the target’s motion state. Mahalanobis distance, measuring the discrepancy between the prediction box and the detection box, serves as the target’s motion feature. In contrast, the cosine distance, which assesses the similarity between the detection box feature vector and the track feature set, defines the target’s appearance feature. These features are then cascaded for matching. For tracks and detection boxes that do not match, the cost matrix is determined by the Intersection Over Union (IOU) value between the prediction and detection boxes. Subsequently, these elements are re-matched by employing the Hungarian algorithm.

The Kalman filter algorithm serves as an optimization tool for estimating the states of dynamic systems. When tracking green peppers, this algorithm predicts the fruit’s motion state in the next frame. This algorithm operates in two phases: Prediction and Update. In the Prediction phase, the motion state of the green pepper at time T is forecasted based on its state at T-1. The Update phase involves a weighted analysis of the green pepper’s motion states at times T-1 and T, culminating in an adjusted motion state at time T. The specific formula is shown in (1)–(6).

Prediction stage:


(1) $$\text{State prediction equation}:{\hat{X}}_{t/t-1}=F_{t}*\;X_{t-1/t-1}+B_{t}*u_{t}$$

(2) $$\text{Covariance prediction equation}:P_{t/t-1}=F_{t}*P_{t-1/t-1}*F_{t}^{T}+Q_{t}$$

${\hat{X}}_{t/t-1\;}$ and $P_{t/t-1}$ are the states and covariance matrices obtained by the prediction; $X_{t-1/t-1}$ is the time state of t-1; $F_{t}$ is the state transition matrix; $u_{t}$ is the external control input; $P_{t-1/t-1}$ is the covariance matrix of the current state; $Q_{t}$ is the covariance matrix of process noise.

Update stage:


(3) $$\text{Measurement update equation}:y_{t}=Z_{t}-H_{t}*{\hat{X}}_{t/t-1\;}$$

(4) $$\text{Kalman gain equation}:K_{t}=P_{t/t-1}*H_{t}^{T}*{\left(H_{t}*P_{t/t-1}*H_{t}^{T}+R_{t}\right)}^{-1}$$

(5) $$\text{Status update equation}:X_{t/t}=\;{\hat{X}}_{t/t-1\;}+K_{t}*y_{t}$$

(6) $$\text{Covariance update equation}:P_{t/t}=\left(I-K_{t}*H_{t}\right)*P_{t/t-1}$$

$y_{t}$ is the measurement residual; $Z_{t}$ is the measured value; $H_{t}$ is the measurement matrix; $R_{t}$ is the covariance matrix of measurement noise**;** $K_{t}$ is the Kalman gain; *I* is the identity matrix; $X_{t/t}$ is the update status at t time.

The Hungarian algorithm, an optimization tool, addresses the optimal allocation problem through combinatorial matching. Within the DeepSort framework, the Hungarian algorithm computes the minimum cost between object detection boxes and tracking tracks, thus determining the most effective matching scheme. The Intersection Over Union (IOU) value between the prediction box and the detection box forms the cost matrix. The Hungarian algorithm then utilizes this matrix to ascertain the optimal match between target detection and tracking. To circumvent ID switching of the detected target, which can occur due to occlusion or overlap, both the motion and appearance features of the target are cascaded for matching.

### 2.3 Improving DeepSort algorithm

Green pepper tracking faces significant challenges due to fruit overlap and branch occlusion. Camera movement, fruit overlap, and branch occlusion can substantially alter green peppers’ motion features between video frames. This alteration hinders the ability to match them with their previous tracks, often resulting in the assignment of new IDs. To mitigate ID switching attributable to changes in green peppers’ motion features, this study integrates an appearance matching algorithm for green peppers into the DeepSort algorithm, thereby enhancing the influence of the matching mechanism based on green pepper appearance features.

**Figure 6:**
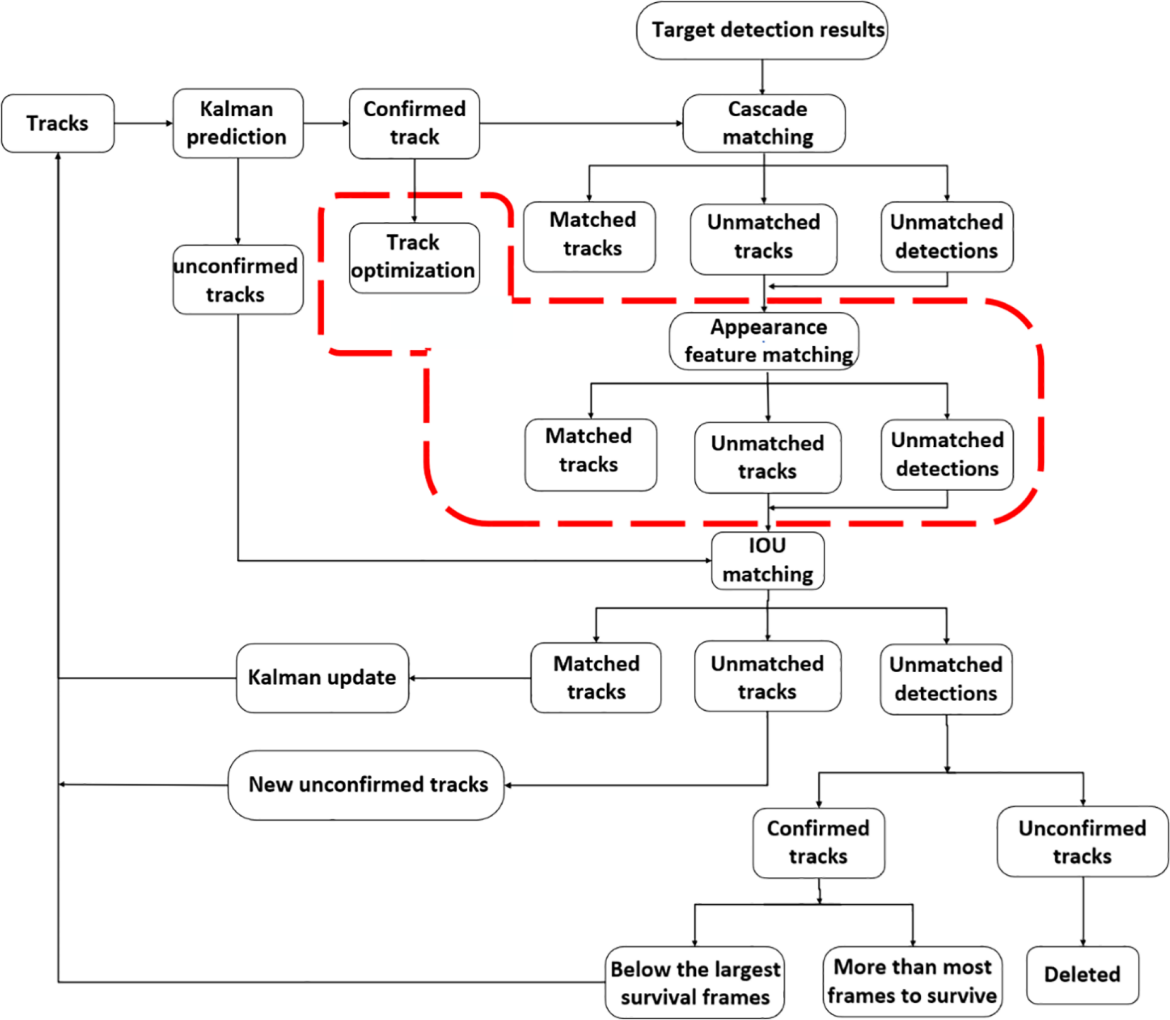
Improved flow chart of DeepSort multi-target tracking algorithm.

Furthermore, to decrease the frequency of ID switching during tracking, the track optimization algorithm from SportsTrack has been incorporated. This method processes the generated tracks by analyzing their correlations, thereby reducing mismatches during tracking. **Figure 6** presents the flow chart of the enhanced DeepSort multi-target tracking algorithm.

#### 2.3.1 Appearance feature matching

**Figure 7:**
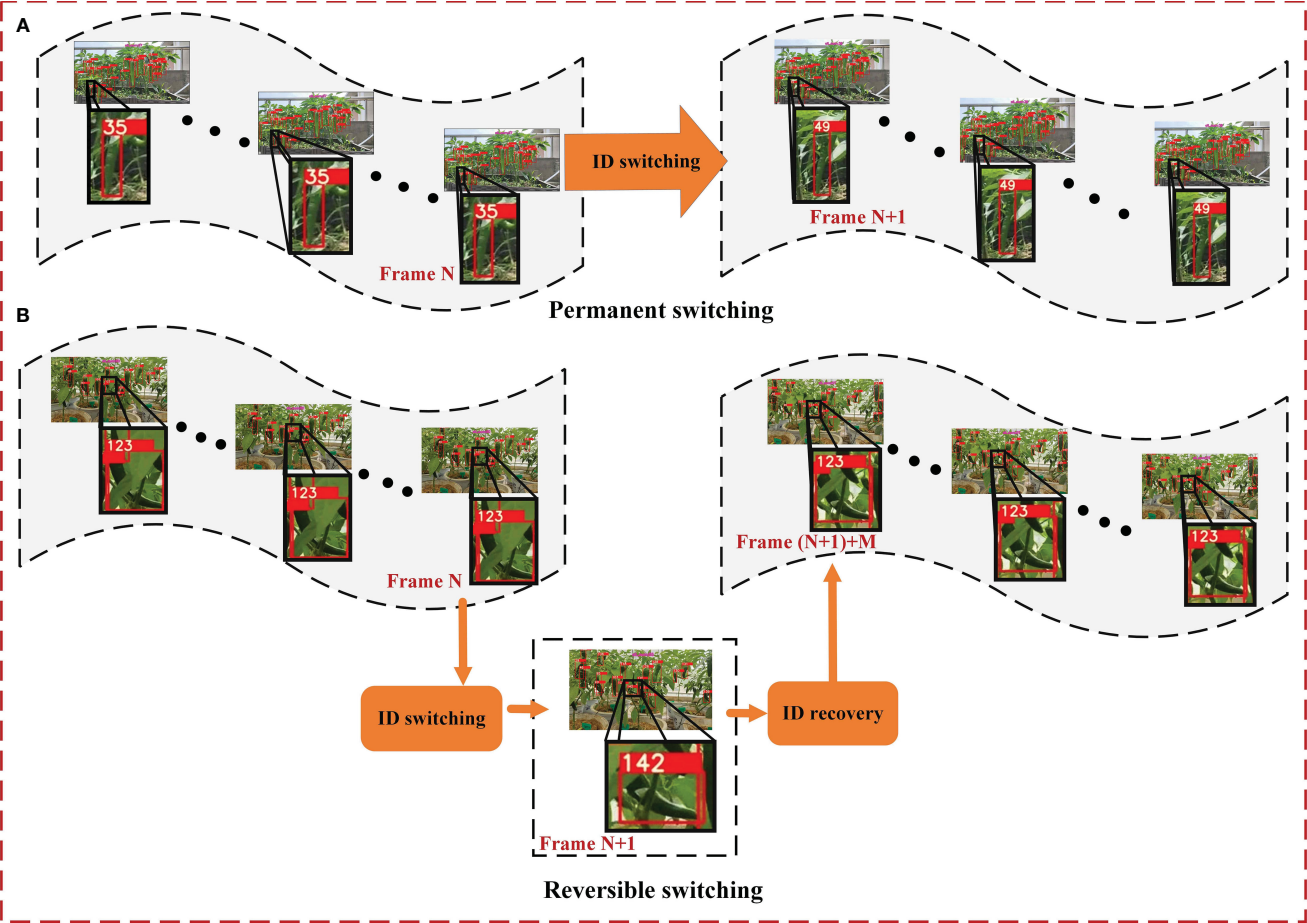
Two cases of green pepper ID switching. **(A)** indicates that the previous ID of the pepper track can no longer be restored after the switch due to the change of pepper motion characteristics, and **(B)** indicates that the previous ID of the pepper track can be restored after the switch due to the change of pepper motion characteristics.

In green pepper fruit tracking, ID switching primarily occurs due to motion feature changes across two video frames, manifesting in two forms: permanent and reversible switching. Permanent switching occurs when the motion features of a green pepper fruit change irrecoverably, leading to the loss of original features and subsequent matching to a new, stable track. This results in a permanent change in the ID of the green pepper fruit, as illustrated in **Figure 7A**. As illustrated in the figure, the green pepper initially identified as ID 35 undergoes a permanent ID switch, changing from 35 to 49. Conversely, reversible switching happens when the motion features of a green pepper fruit mutate but swiftly recover, allowing the original features to persist and the initial track to be re-associated. In such instances, the green pepper fruit’s ID undergoes a reversible change, as depicted in **Figure 7B**. As can be seen from the figure, the green pepper fruit with ID 123 briefly switches to 142 and then returns to 123. However, for the green pepper count, ID 142 will be recorded. In both scenarios, the green pepper fruit risks being double-counted.

The cascade matching within the DeepSort algorithm facilitates the alignment between detected green peppers and generated tracks, following a weighted assessment of both appearance and motion features. Nevertheless, when motion features undergo significant changes, cascade matching may fail, leading to the generation of a new track and necessitating the re-matching of the detected green pepper fruit. This study enhances the matching mechanism of the DeepSort algorithm, amplifying the role of green pepper appearance features in the target-track association process. Post-cascade matching, an additional green pepper appearance feature matching algorithm is incorporated. This step re-matches green peppers and tracks that become dissociated due to motion feature mutations, thereby reducing the incidence of ID switching in green pepper tracking.

#### 2.3.2 Track optimization

**Figure 8:**
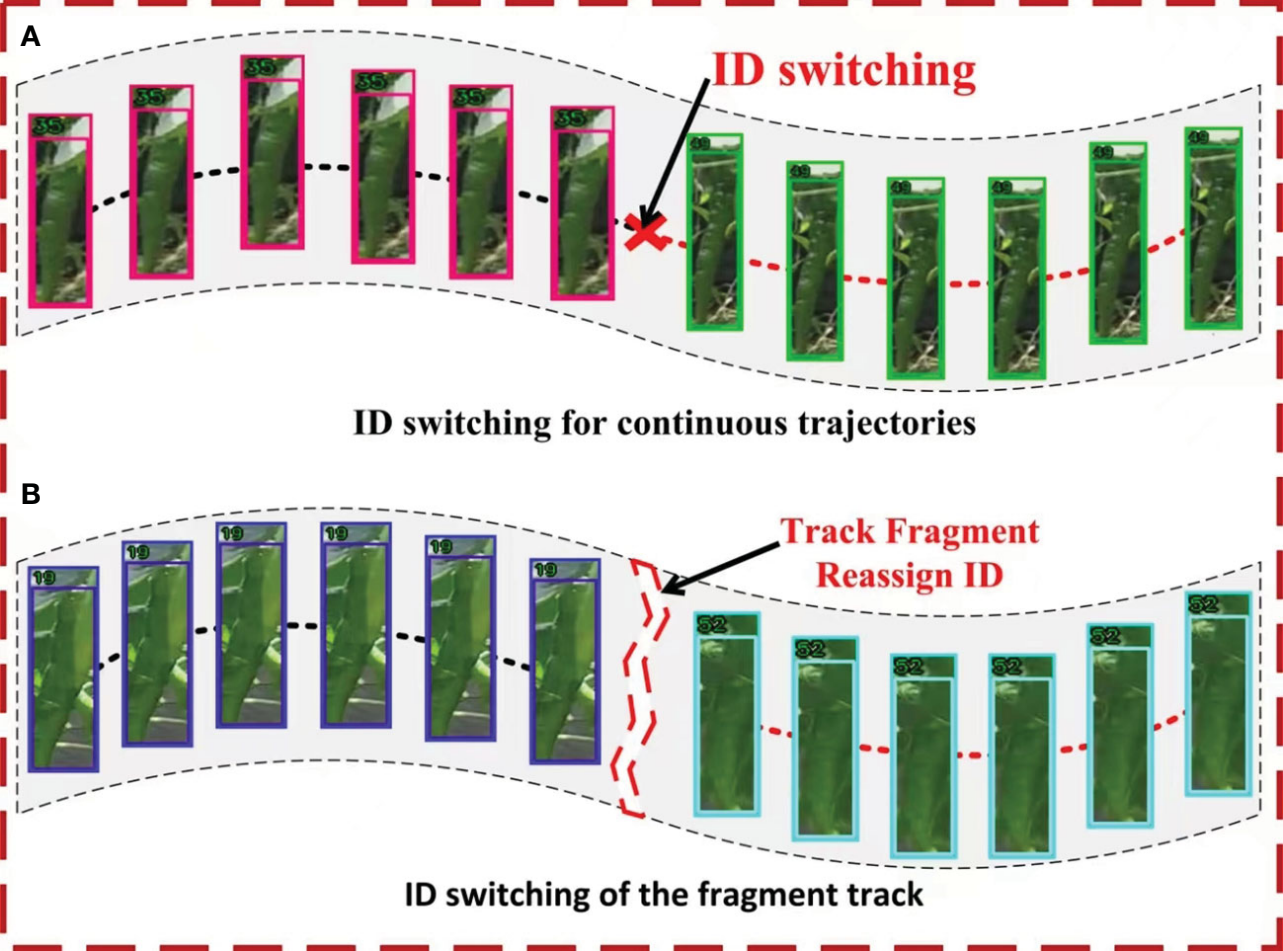
Track optimization of two cases. **(A)** represents ID switching in the continuous track, and **(B)** represents ID switching in the pepper track in the track fragment.

The optimization of tracking can be broadly classified into two primary categories: continuous track optimization and fragment track optimization. As shown in **Figure 8A**, in a continuous time series, multiple tracks are successively linked to the same green pepper, resulting in an ID switch for the fruit. In fragment track optimization, illustrated in **Figure 8B**, occlusions or camera movements cause a green pepper to temporarily disappear and reappear, matching with a new track and resulting in an ID switch in fragmented time. In both scenarios, there is a risk of double-counting the green pepper fruit. The improved SportsTrack track optimization algorithm is applied to refine green pepper tracking, aiming to minimize this double-counting error.

In the captured video sequence, each green pepper fruit exhibited uniform linear motion and possessed a distinct shape, markedly differing from the athletes tracked in SportsTrack (Wang et al., 2023). This study integrates position and shape feature matching into the SportsTrack track optimization algorithm, enhancing the accuracy of the optimization process. Initially, unstable and partially stable tracks are eliminated based on the variance in their appearance feature similarity. For two stable track segments containing M and N frames, respectively, a similarity matrix is computed if they persist for a reasonable duration. Should the count of M×N similarity values exceed half of their product, the position and shape features of the two tracks are matched. Upon satisfying all criteria, the two tracks are deemed to have the same ID and consequently merged into a single track. **Supplementary Figure 2** illustrates the complete track optimization process.

## 3 Experimental

### 3.1 Dataset acquisition

The green pepper variety utilized in this experiment was ‘Xiangyan 15’, cultivated in the experimental field of Hunan Agricultural University, located in Furong District, Changsha City, Hunan Province. When collecting data, the self-made radio-controlled car was driven at a constant speed using a remote control., A DJI Osmo Mobile SE handheld stabilizer was mounted on the car to secure the phone and minimize vibrations, ensuring stable image capture. The phone model used was an OPPO Reno6 Pro+. The specific parameters of the remote-controlled car are listed in **Table 1**.

**Table 1:** The parameters of the radio-controlled car.

Parameter	Value/Method
Drive mode	Four-wheel drive
Speed range	0–2m/s
Steering control	Differential steering
Load capacity	0–5kg

At 8 am (low), 12 noon (high), and 4 PM (middle) different light intensities, the radio-controlled car at 0.2m/s speed to collect data. The fruit density was distinguished according to the number of green peppers in each image. **Figure 1** illustrates the shooting process and the resulting image quality. The dataset comprises 1200 images, each with a resolution of 1920×1080, stored in JPG format. Additionally, seven videos were captured at a resolution of 1920×1080, in MP4 format, with a frame rate of 30 fps. Detailed information about each video is provided in **Table 2**.

**Table 2:** Detailed information about each video.

	Video	Light intensity	Green pepper density	Video duration/s
Test	Video001	Medium	Low	12
	Video002	Medium	High	17
	Video003	Low	High	20
Train	Video004	High	Low	6
	Video005	Medium	Medium	11
	Video006	Medium	Medium	17
	Video007	Low	Medium	27

To augment the green pepper image dataset, standard data enhancement techniques including Image Rotation, Gaussian Blur, Mirror Flip, and Brightness Change were employed in random combinations. The effects of this image expansion are depicted in **Supplementary Figure 3**. Consequently, the green pepper image dataset expanded from 1200 to 4800 images. The data set is divided into the training set and the test set by a ratio of 7:3. Similarly, the video dataset was split into training and test datasets in a 4:3 ratio. This division was designed to facilitate the training of the ReID model within the DeepSort algorithm and to evaluate its tracking performance and counting accuracy.

### 3.2 Experimental platform

The experimental setup utilized an HP DELL T5820 workstation, configured with a XEON W2155 CPU, 32GB of RAM, and an NVIDIA RTX2080Ti 12G graphics card. The Pytorch1.8 deep learning framework was deployed on a Windows 10 operating system, with Python 3.8 as the programming language.

For YOLOv5s model training, input images were resized to 640×640 pixels. Stochastic Gradient Descent (SGD) was employed to optimize the model parameters. The Weight Decay was set at 0.005, with a Momentum Factor of 0.937. The training used a Batch Size of 4 across 300 Epochs.

During ReID model training, the ResNet network served as the base model. Input images were resized to 128×256, with Stochastic Gradient Descent (SGD) and a Momentum Factor of 0.9 for optimization. Leveraging Market’s official pre-training weights, the training was conducted with a Batch Size of 64 over 300 Epochs.

### 3.3 Evaluation metrics

In assessing the object detection algorithm, key metrics include Recall (R), Precision (P), mean Average Precision (mAP), and the Detection Time for a single image and GFLOPs. These metrics are defined by the formulas in Equations (7)–(9).


(7) $$Precision=\;\frac{TP}{TP+FP}$$

(8) $$Recall=\;\frac{TP}{TP+FN}$$

(9) $$mAP=\;\frac{\sum_{k=0}^{n}AP}{N}$$

Here: TP - the number of real samples;

FP - the number of false positive samples;

FN – number of false negative samples;

N - the number of classes in the sample.

The evaluation of the multi-target tracking algorithm employs ID Switch (IDs) (Luo et al., 2014), Multiple Object Tracking Accuracy (MOTA), and Multiple Object Tracking Precision (MOTP) as key metrics. IDs quantifies the frequency of ID changes for the same target caused by incorrect associations; ideally, this metric should be zero. MOTA effectively gauges the performance of tracking algorithms in terms of target detection and track stability. MOTP assesses the positioning accuracy of the detection system. The calculations for MOTA and MOTP are detailed in Equations (10) and (11).


(10) $$MOTA=1-\frac{\sum FP+FN+IDSW}{\sum GT}$$

(11) $$MOTP=\;\frac{\sum_{t,i}d_{t,i}}{\sum_{t}c_{t}}\times 100\%$$

Here: FN - the number of false negative examples;

FP - the number of false positive examples;

GT - the number of labeled targets;

IDSW - the number of ID switch;

$c_{t}$ - the number of successful matches in the current frame;

$d_{t,i}$ - the distance between the real box and the detection box;

i - the current detection target;

t - the sequence number of the frame.

To evaluate the performance of green pepper counting in video sequences, three indexes are proposed: Average Counting Precision (ACP), Mean Absolute Error (MAE), and Root Mean Square Error (RMSE). ACP and MAE can measure the performance of the model and reflect the accuracy of counting, while RMSE can reflect the robustness of the model (Jiang et al., 2021). These metrics assess both the accuracy and error in counting. The formulas for these evaluation indexes are provided in Equations (12)–(14).


(12) $$ACP=\;\frac{\sum_{1}^{N}\left(1-\frac{\left|GT-COUNT\right|}{GT}\right)}{N}$$

(13) $$MAE=\;\frac{\sum_{1}^{N}\left|GT-COUNT\right|}{N}$$

(14) $$RMSE=\;\sqrt{\frac{\sum_{1}^{N}{\left(GT-COUNT\right)}^{2}}{N}}$$

Here: GT - the number of labeled targets;

COUNT - the number of statistics;

N - the number of videos.

## 4 Results analysis

### 4.1 Detection results and analysis

The training process of the model is depicted in **Supplementary Figure 4**, showing that the CS_YOLOv5s model converges faster during training, with a lower final loss value compared to YOLOv5s. To evaluate the impact of each improvement module on the YOLOv5s model, ablation experiments were conducted on the test set, and the results are presented in **Table 3**.

**Table 3:** Test comparison of test results.

Baseline model	Slim-Nick +GSConv	CBAM	P/%	R/%	mAP/%	Detection time of a single image/ms	GFLOPs
YOLOv5s			99	93.9	96.8	9.4	16.3
YOLOv5s	√		98.7	94.5	96.9	6.0	15.3
YOLOv5s	√	√	98.6	95	97.3	6.1	15.4

“√” indicates the addition of this module based on the YOLOv5s model.

After applying the Slim-Neck + GSConv structure to the YOLOv5s model, the precision decreased, but recall and mAP values increased. Additionally, the Detection time of a single image and GFLOPs were reduced. Further adding the CBAM attention mechanism resulted in the proposed CS_YOLOv5s model. Experiment results show that with the addition of the CBAM attention mechanism, which enhances the model’s attention to green pepper fruit features, recall and mAP values improved, while precision, detection time of a single image, and GFLOPs remained relatively unchanged. Comparing the YOLOv5s model with the CS_YOLOv5s model, the latter exhibits slightly lower accuracy but a 1.1% increase in recall, a 0.5% increase in mAP value, and a reduction of 3.3ms in the detection time per single image.

**Figure 9:**
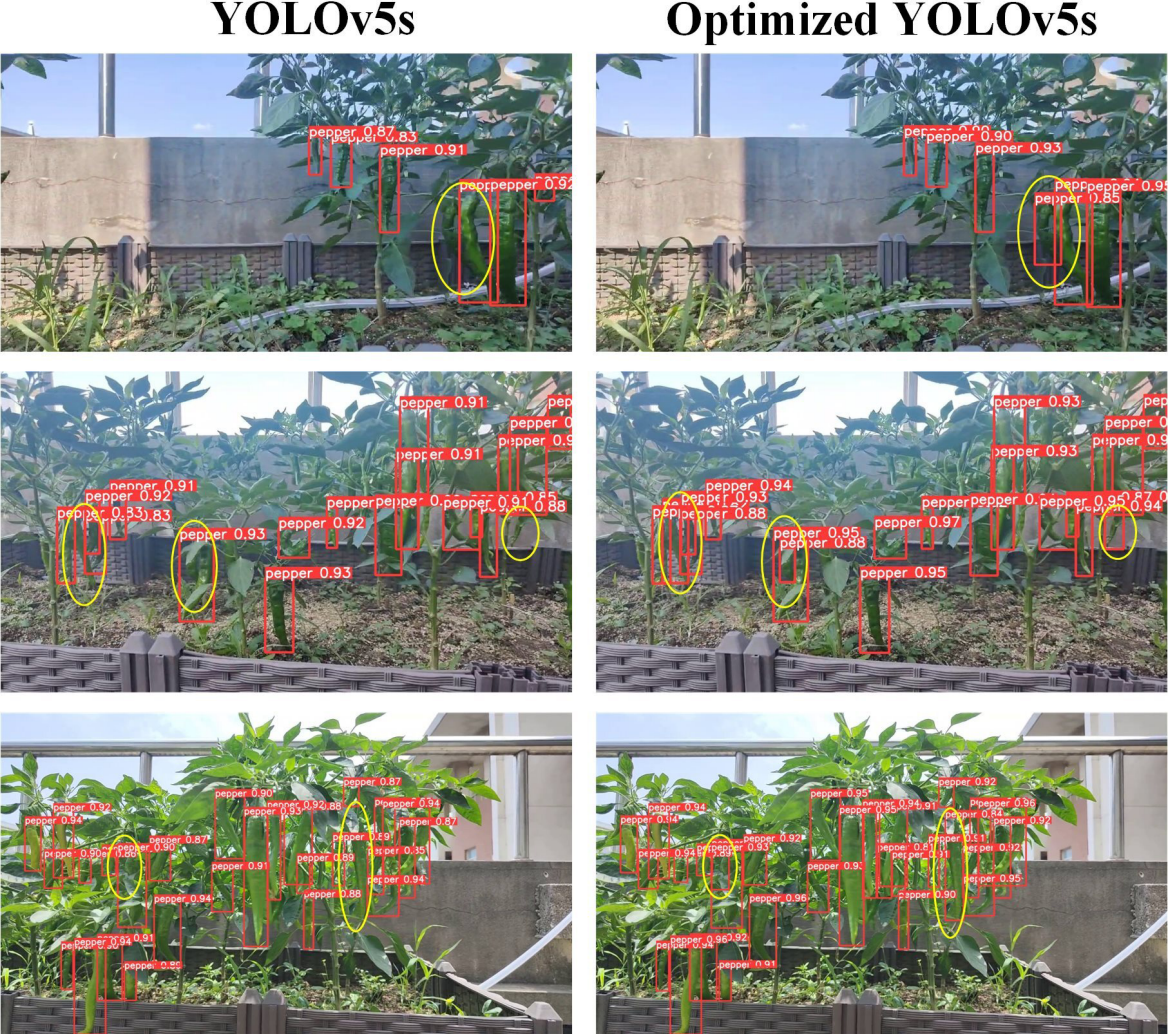
Detection results of different density models.

The detection results of the model for green peppers with different densities are shown in **Figure 9**. As illustrated in **Figure 9**, the optimized YOLOv5s algorithm demonstrates superior detection confidence for green peppers across different densities, compared to the standard YOLOv5s algorithm. Furthermore, the optimized YOLOv5s algorithm notably reduces missed detections of green pepper, thereby enhancing the stability of subsequent green pepper tracking.

This paper compares CS_YOLOv5s with the YOLO series and the DR_DETR object detection algorithm, as shown in **Table 4**. From the table, it is evident that YOLOv5s has a significant advantage in recognizing chili peppers. Although YOLOv6s, YOLOv8s, and YOLOv3-tiny exhibit slightly higher Precision than YOLOv5s, their Recall and mAP values are much lower than YOLOv5s, and they have higher parameter counts. While RT-DETR achieves good performance, its parameter count is excessively large. The proposed CS_YOLOv5s in this paper achieves good levels of recall rate, precision rate, mAP value, and GFLOPs, meeting the requirements for chili pepper recognition and subsequent tracking tasks.

**Table 4:** Comparison of multiple target detection algorithms.

model	P/%	R/%	mAP/%	GFLOPs
CS_YOLOv5s	98.60	95.00	97.30	15.40
YOLOv5s	99.00	93.90	96.80	16.30
RT-DETR	95.80	96.80	97.90	108.30
YOLOv3-tiny	99.00	78.00	88.00	19.00
YOLOv6s	99.00	90.80	95.20	44.20
YOLOv8s	99.20	91.40	95.50	28.40

### 4.2 Track results and analysis

This experiment incorporates both the appearance feature matching algorithm and the track optimization algorithm into the DeepSort framework. To evaluate the impact of each component on the DeepSort algorithm, CS_YOLOv5s was used as the object detector, and the two algorithms were tested in turn.

Following the integration of the two algorithms into the DeepSort framework, the tracking effectiveness of green pepper fruit under three distinct conditions was compared, with the results detailed in **Table 5**. The test results indicate that post-integration, the Multiple Object Tracking Accuracy (MOTA) and Multiple Object Tracking Precision (MOTP) for green pepper tracking under various conditions exhibit minimal fluctuations, while the number of ID switches is notably reduced. Videos 001 and 002 share identical lighting conditions but differ in green pepper density. A comparative analysis of their test results reveals the effectiveness of both algorithms in track optimization, with the addition of the appearance feature matching algorithm showing a more pronounced impact. Videos 002 and 003 present the same green pepper density, yet Video 003 features lower light intensity. The comparison between these videos indicates an enhanced optimization effect from the appearance feature matching algorithm under reduced light conditions. These results suggest that lower light intensity leads to increased correlation errors between the target and track, attributable to changes in the motion features of green pepper fruit.

**Table 5:** Test comparison of tracking effect.

model	Name	Algorithm	GT	MOAT/%	MOTP/%	IDs
CS_YOLOv5s	Video 001	Deep Sort	57	91.0	85.9	15
		Deep Sort + appearance matching		91.2	85.9	14
		Deep Sort + optimization		91.0	85.9	15
		Deep Sort + appearance matching +optimization		92.2	86.1	14
	Video 002	Deep Sort	95	92.7	86.8	29
		Deep Sort+ appearance matching		92.8	86.8	23
		Deep Sort+ optimization		92.7	86.8	27
		Deep Sort + appearance matching+ optimization		92.8	87.4	21
	Video 003	Deep Sort	96	88.6	88.3	24
		Deep Sort+ appearance matching		89.4	88.0	14
		Deep Sort+ optimization		88.7	88.3	23
		Deep Sort + appearance matching + optimization		89.4	87.3	13

Upon comprehensive analysis, it was observed that the standalone integration of the appearance feature matching algorithm resulted in a 25% reduction in the number of ID switches. Furthermore, the exclusive addition of the track optimization algorithm led to a 4.41% decrease in ID switches. When both the appearance feature matching and track optimization algorithms were combined, there was a notable reduction of 29.41% in ID switching. These results are detailed in **Table 6**.

**Table 6:** Comprehensive comparison of tracking effect test.

model	Numberof video	Algorithm	GT	MOAT/%	MOTP/%	IDs
CS_YOLOv5s	3	Deep Sort	247	90.9	87.1	68
		Deep Sort+ appearance matching		91.3	86.9	51
		DeepSort+ optimization		90.9	87.1	65
		DeepSort + appearance matching + optimization		91.3	86.9	48

### 4.3 Counting results and analysis

In order to explore the influence of the appearance matching algorithm and track optimization algorithm on the counting of green pepper fruit, CS_YOLOv5s was used as the detector of DeepSort algorithm to carry out the counting comparison experiment of green pepper fruit. The count of green pepper fruits under three distinct conditions within the test set, as detailed in **Table 1**, was comparatively analyzed, with the findings presented in **Table 7**. An examination of the test results from Videos 001 and 002 reveals that an increase in green pepper fruit density correlates with a decrease in counting accuracy and an increase in missed detections. This trend is attributed to the intensified influence of green pepper branches, leaves, and fruit overlap on both detection and tracking as the density increases. Comparing Videos 002 and 003, a decrease in light intensity is observed to lead to lower counting accuracy and higher missed detection rates for green pepper fruit. The difficulty in identifying green pepper fruit in low-light conditions is the primary reason for this outcome. Upon comparing the three scenarios, it is evident that under challenging conditions of low light and high-density green pepper fruit, the integration of both algorithms significantly enhances counting accuracy.

**Table 7:** Comparison of counting effect tests.

Video	Algorithm	GT	Count	IDSW	ACP	MAE
Video 001	DeepSort	57	63	15	89.47%	6
	DeepSort+ appearance matching		62	14	91.23%	5
	DeepSort + optimization		63	15	89.47%	6
	DeepSort + Appearance matching + optimization		62	14	91.23%	5
Video 002	DeepSort	95	104	29	90.52%	9
	DeepSort+ appearance matching		99	23	95.79%	4
	DeepSort + optimization		100	27	94.74%	5
	DeepSort + Appearance matching + optimization		96	21	98.95%	1
Video 003	DeepSort	96	111	24	84.38%	15
	DeepSort+ appearance matching		102	14	93.75%	6
	DeepSort + optimization		108	23	87.50%	12
	DeepSort+ Appearance matching + optimization		100	13	95.83%	4

A comprehensive evaluation across all three scenarios was conducted to compare the effects of the two algorithms on counting accuracy, with the findings detailed in **Table 8**. In the absence of optimization algorithms, the Average Counting Precision (ACP) stands at 88.13%, the Mean Absolute Error (MAE) at 10, and the Root Mean Square Error (RMSE) at 10.68. Following the sequential addition of the appearance feature matching and track optimization algorithms, the ACP increases by 5.46% and 2.44% respectively, while both the MAE and RMSE decrease correspondingly. When both algorithms are integrated into the DeepSort algorithm simultaneously, for green pepper counting in videos, the ACP (Average Counting Precision), MAE (Mean Absolute Error), and RMSE (Root Mean Squared Error) values of 95.33%, 3.33, and 3.74, respectively. Compared to the original algorithm, ACP increases by 7.2%, while MAE and RMSE decrease by 6.67 and 6.94, respectively. The integration of these two algorithms results in a substantial enhancement of the counting accuracy robustness of the algorithm.

**Table 8:** Comprehensively comparison of counting effect tests.

Algorithm	Number of videos	ACP	MAE	RMSE
DeepSort	3	88.13%	10	10.68
DeepSort + appearance matching	3	93.59%	5	5.07
DeepSort + optimization	3	90.57%	7.67	8.27
DeepSort+ Appearance matching + optimization	3	95.33%	3.33	3.74

To investigate the impact of YOLOv5s and CS_YOLOv5s on green pepper fruit counting, comparative experiments were conducted using the improved DeepSort algorithm as the basis. The experimental results are summarized in **Table 9** below. In conclusion, the CS_YOLOv5s model demonstrates significant advantages in green pepper fruit counting accuracy and robustness compared to YOLOv5s. However, the FPS improvement when processing videos using CS_YOLOv5s is only slightly higher than that of YOLOv5s, indicating that the matching process while tracking green pepper fruits consumes significant computational resources. Based on this study, the processing speed of videos reaches 17 FPS, which is insufficient for the real-time processing of 30 FPS videos. In order to realize the real-time detection of green pepper fruit, it is necessary to reduce the video frame rate to 15FPS. Obviously, after the frame rate is reduced, the sampling interval of the two frames of images is not consistent with the sampling in this experiment. Therefore, the speed of the radio-controlled car should be reduced by half.

**Table 9:** Effect of target detector on counting.

Algorithm	Model	Number of videos	ACP	MAE	RMSE	FPS
DeepSort + Appearance matching + optimization	YOLOv5s	3	93.29%	5.33	5.58	15.9
	CS_YOLOv5s	3	95.33%	3.33	3.74	16.2

### 4.4 Track effect improvement

#### 4.4.1 Comparison results of adding appearance feature matching

**Figure 10:**
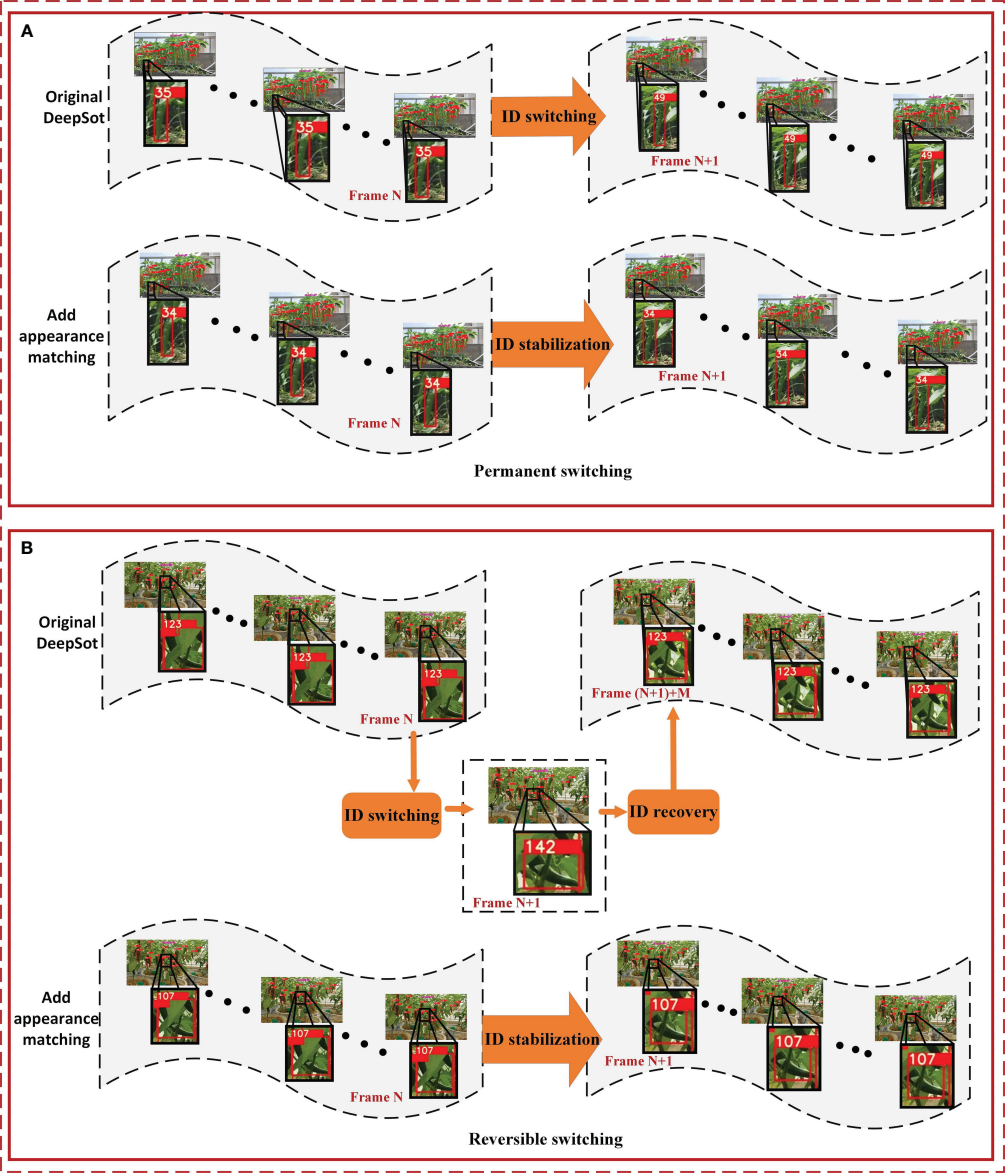
Effect of green pepper tracking after improvement. **(A)** shows the improvement effect of pepper trajectory under the condition of 'A' in **Figure 7** before and after the algorithm is used; **(B)** shows the improvement of pepper trajectory in the case of 'B' in **Figure 7** before and after the algorithm is used.

In the DeepSort algorithm, the green pepper fruit’s appearance feature matching algorithm was integrated into the matching process, with the test results depicted in **Figure 10**. Post-integration, this algorithm effectively alleviates the two primary types of false associations stemming from the motion feature mutations of green pepper fruit, leading to a reduction in the number of ID switches.

#### 4.4.2 Comparison results of track optimization

**Figure 11:**
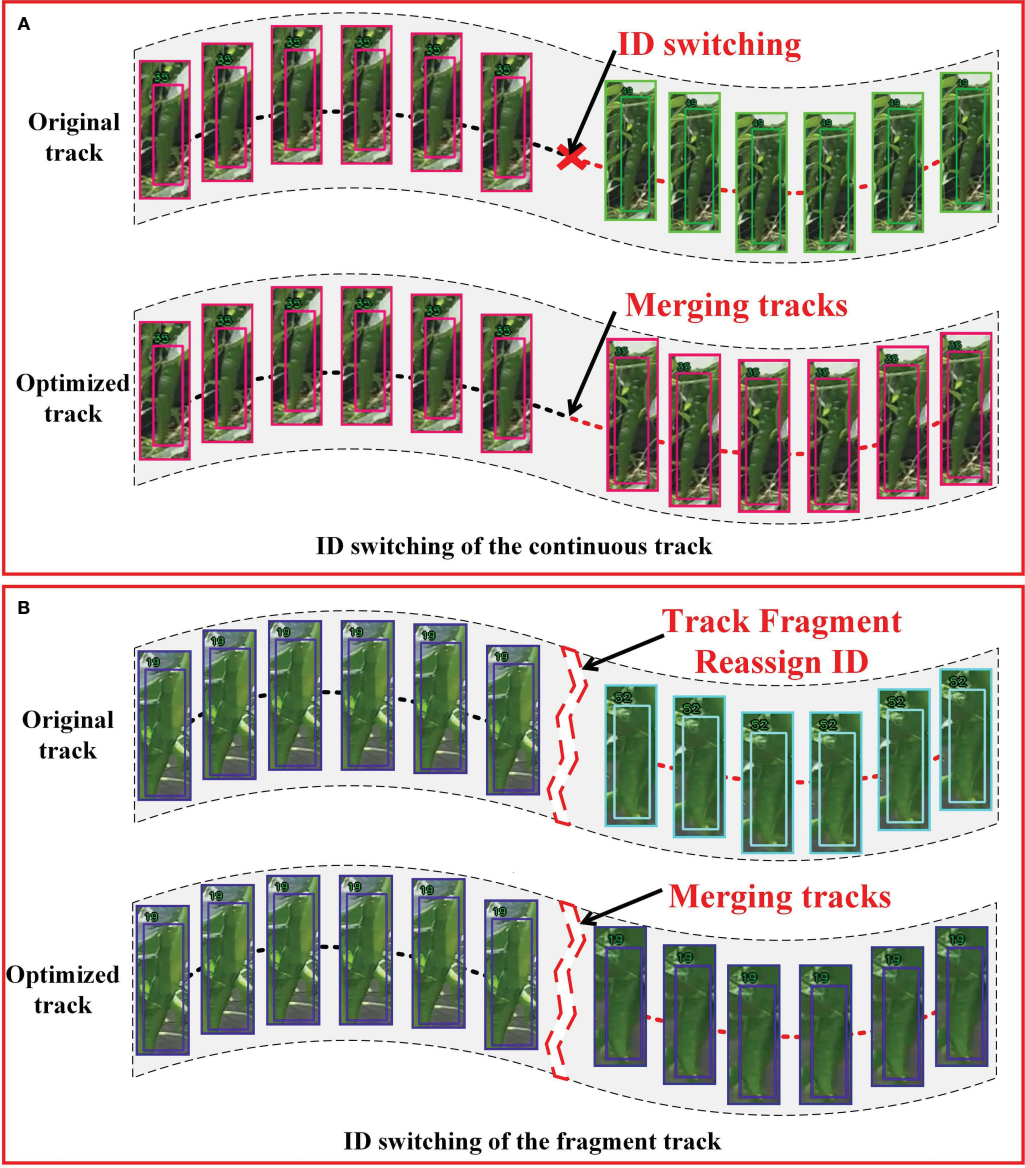
Results of green pepper track optimization. **(A)** shows the improvement effect of pepper track under the condition of 'A' in **Figure 8** before and after the algorithm is used; **(B)** shows the improvement of pepper trajectory in the case of 'B' in **Figure 8** before and after the algorithm is used.

The optimization of the green pepper fruit tracking process significantly alleviated incorrect target-to-track matching, with the test results presented in **Figure 11**. By utilizing track context information, issues of ID switching in both continuous and fragmented tracks were mitigated, resulting in more stable tracking of green pepper fruit.

## 5 Conclusion

This paper focuses on the fusion and individual optimization of the DeepSort multi-target tracking algorithm and the YOLOv5s target detection algorithm to enable accurate counting of green peppers in video sequences, conducting experiments to verify the efficacy of these optimizations. The experiments led to the following conclusions:

(1) A target detection model named CS_YOLOv5s based on the YOLOv5s framework was proposed. CS_YOLOv5s utilizes a Slim-Neck combined with GSConv to optimize the Neck layer, balancing the model’s detection accuracy and speed. Additionally, the model incorporates the CBAM attention mechanism to enhance the feature perception capability of the model for different positions of green pepper fruits, thereby improving feature extraction. Experimental results demonstrate that the CS_YOLOv5s model achieved an mAP (mean Average Precision) of 98.96%, Precision rate of 95%, Recall rate of 97.3%, and Detection Time for a single image of 6.3 ms. Comparative analysis under different densities of green pepper fruits showed that the performance of the CS_YOLOv5s model surpassed that of the original model.
(2) In response to the causes of ID switching observed in green pepper fruit tracking, this paper enhances the matching mechanism of the DeepSort algorithm, placing greater emphasis on green pepper characteristics during the matching process. Additionally, the track optimization algorithm from SportsTrack was employed and refined for improved green pepper track. Using CS_YOLOv5s as the object detector, after sequentially integrating these enhanced functionalities into the DeepSort algorithm, while the improvements in MOAT (Multiple Object Tracking Accuracy) and MOTP (Multiple Object Tracking Precision) are modest, a significant reduction in ID switching is observed. By simultaneously integrating these two algorithms into DeepSort, the number of ID switches for pepper fruits in the video decreased from 68 to 28, representing a reduction of 29.41%.
(3) The impact of appearance feature matching and track optimization algorithms on the counting accuracy of green pepper fruit was experimentally investigated within the DeepSort framework. These two algorithms demonstrated varying degrees of effectiveness in enhancing the counting accuracy of green peppers. When compared across various environments, the appearance feature matching algorithm was found to more effectively improve counting accuracy. After integrating both algorithms, the Average Counting Precision (ACP) increased to 95.33%, the Mean Absolute Error (MAE) decreased to 3.33, and the Root Mean Square Error (RMSE) decreased to 3.74. Compared to the original algorithm, ACP increases by 7.2%, while MAE and RMSE decrease by 6.67 and 6.94, respectively. The algorithm significantly improves the counting accuracy and robustness of green pepper fruits. and based on the optimized DeepSort algorithm, the influence of different detectors on counting is compared, and it is found that better detectors will get better results.

## 6 Outlook

The research demonstrates that combining object detection algorithms with object tracking algorithms can effectively, quickly, and accurately count pepper fruits, thereby estimating pepper fruit yield, which has practical value. However, there are numerous varieties of peppers, and harvesting requirements and growing conditions vary. Therefore, future research will further expand to acquire more datasets to enhance adaptability to different agricultural environments.

In addition, the research shows that the fruit counting of green pepper can basically achieve real-time detection, but the real-time detection frame rate needs to be improved by further optimization algorithms. The tracking and matching process of pepper fruit consumes a lot of time and computing resources. Future efforts could focus on optimizing matching algorithms to simplify them and improve matching speed while ensuring accuracy.

## 7 Declaration of generative AI and AI-assisted technologies in the writing process

Statement: During the preparation of this work the author(s) used ChatGPT4 in order to improve readability and language. After using this tool/service, the author(s) reviewed and edited the content as needed and take(s) full responsibility for the content of the publication.

## Data availability statement

The original contributions presented in the study are included in the article/**Supplementary Material**. Further inquiries can be directed to the corresponding author.

## Author contributions

PD: Conceptualization, Data curation, Formal analysis, Investigation, Methodology, Project administration, Resources, Software, Validation, Visualization, Writing – original draft, Writing – review & editing. YX: Data curation, Funding acquisition, Project administration, Resources, Software, Supervision, Validation, Writing – review & editing. SC: Formal analysis, Methodology, Supervision, Writing – review & editing. XL: Conceptualization, Formal analysis, Supervision, Writing – review & editing. WH: Data curation, Supervision, Writing – review & editing. NL: Methodology, Project administration, Supervision, Writing – review & editing. XML: Software, Supervision, Writing – review & editing.
