# Supplementary figures and images for: Green pepper fruits counting based on improved DeepSort and optimized Yolov5s
Source: Front Plant Sci. 2024 Jul 16;15:1417682. doi: 10.3389/fpls.2024.1417682 (PMC11286429; doi:10.3389/fpls.2024.1417682)

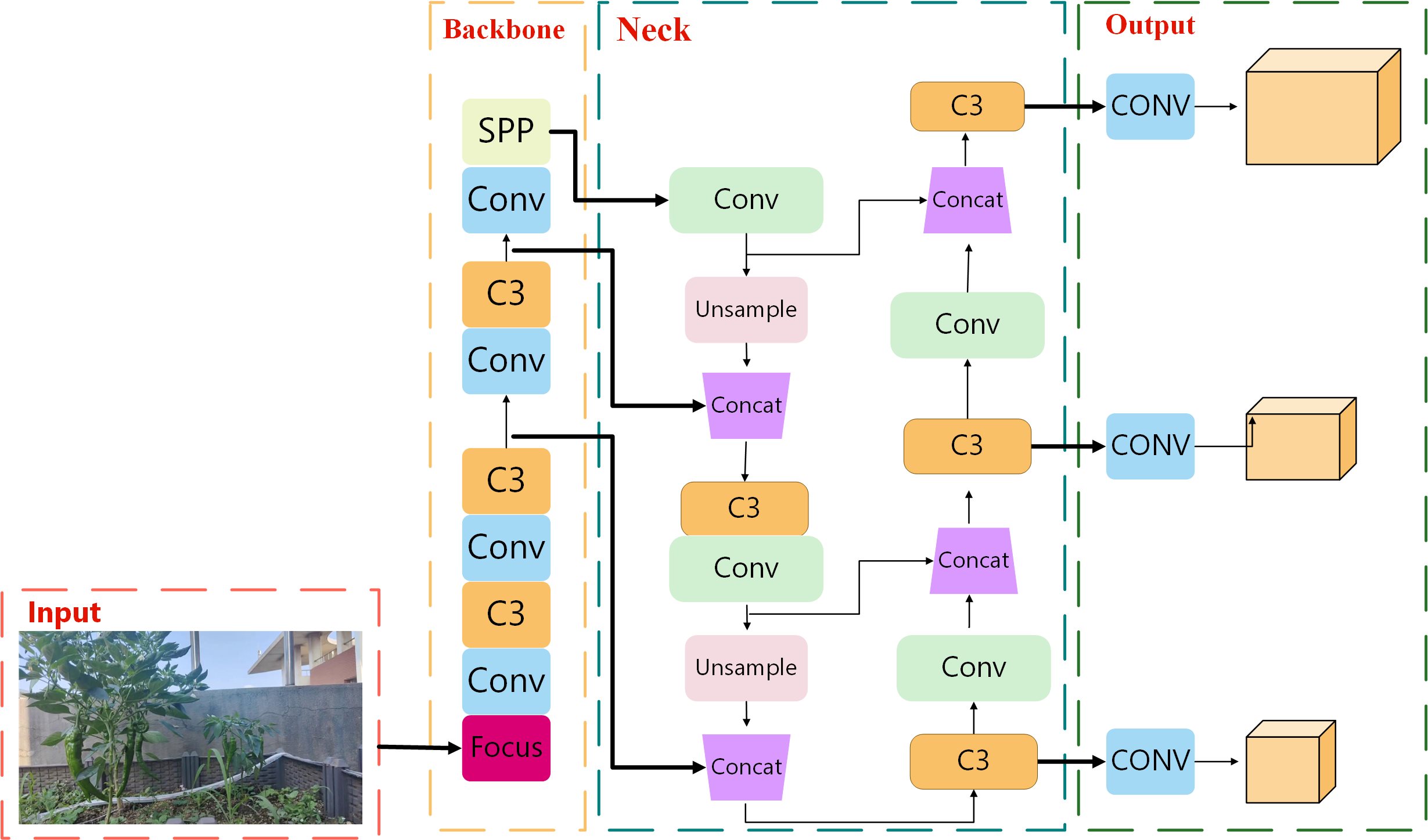

Supplement: Supplementary file 1 [file Image_1.jpeg]

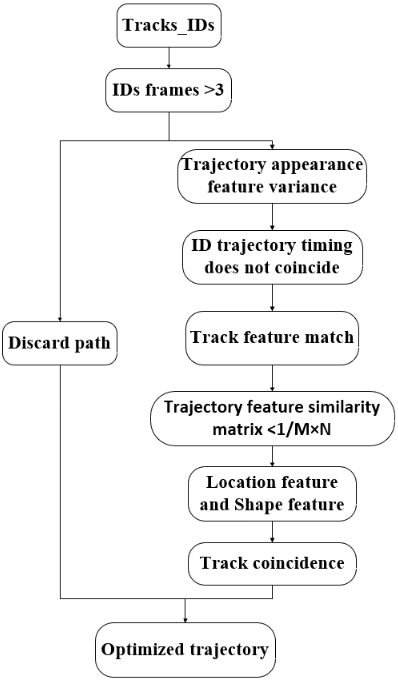

Supplement: Supplementary file 2 [file Image_2.jpeg]

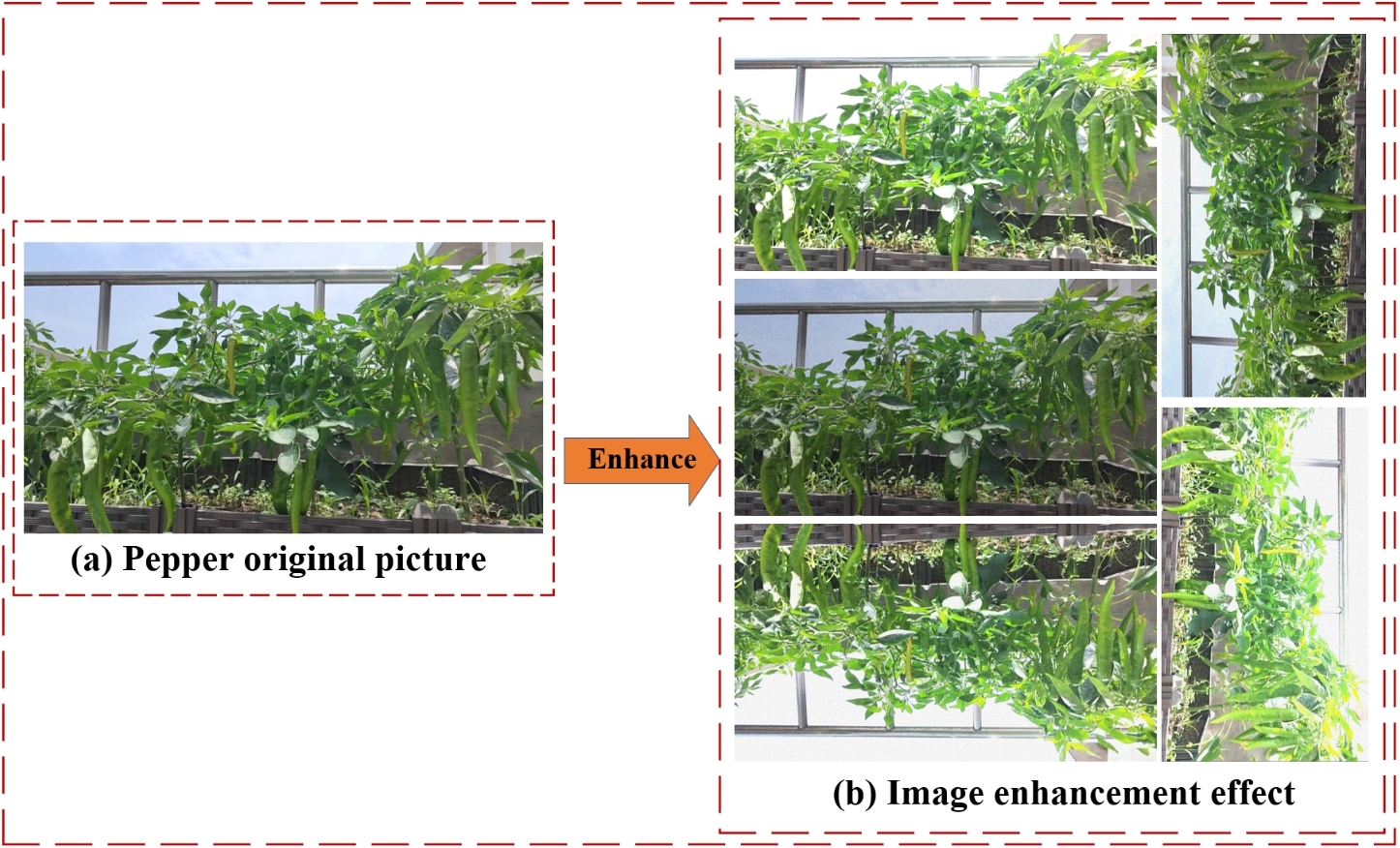

Supplement: Supplementary file 3 [file Image_3.jpeg]

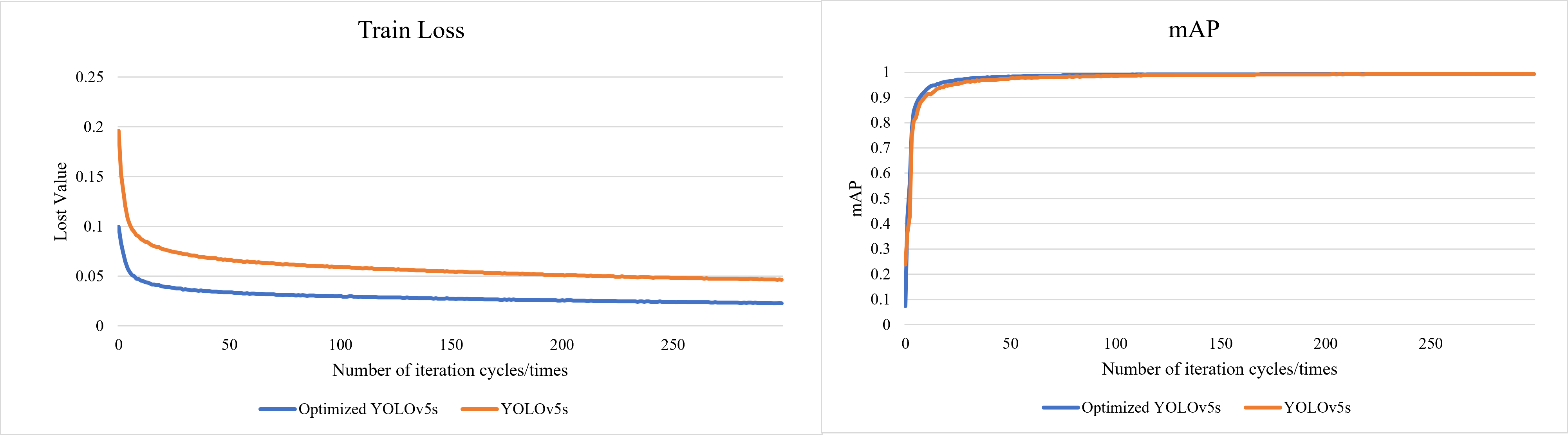

Supplement: Supplementary file 4 [file Image_4.jpeg]
